# Simulation modelling as a tool for knowledge mobilisation in health policy settings: a case study protocol

**DOI:** 10.1186/s12961-016-0143-y

**Published:** 2016-09-21

**Authors:** L. Freebairn, J. Atkinson, P. Kelly, G. McDonnell, L. Rychetnik

**Affiliations:** 1 ACT Health, GPO Box 825, Canberra, ACT 2601 Australia; 2 The Australian Prevention Partnership Centre, Sax Institute, PO Box K617, Haymarket, NSW 1240 Sydney, Australia; 3 School of Medicine, University of Notre Dame, PO Box 944, 2007 Sydney, Australia; 4 Australian National University, Canberra, ACT 2601 Australia; 5 University of New South Wales, Sydney, NSW 2052 Australia

**Keywords:** Health systems, Participatory simulation modelling, Gestational diabetes mellitus, Group model building, Evaluation, Knowledge mobilisation

## Abstract

**Background:**

Evidence-informed decision-making is essential to ensure that health programs and services are effective and offer value for money; however, barriers to the use of evidence persist. Emerging systems science approaches and advances in technology are providing new methods and tools to facilitate evidence-based decision-making. Simulation modelling offers a unique tool for synthesising and leveraging existing evidence, data and expert local knowledge to examine, in a robust, low risk and low cost way, the likely impact of alternative policy and service provision scenarios. This case study will evaluate participatory simulation modelling to inform the prevention and management of gestational diabetes mellitus (GDM). The risks associated with GDM are well recognised; however, debate remains regarding diagnostic thresholds and whether screening and treatment to reduce maternal glucose levels reduce the associated risks. A diagnosis of GDM may provide a leverage point for multidisciplinary lifestyle modification interventions. This research will apply and evaluate a simulation modelling approach to understand the complex interrelation of factors that drive GDM rates, test options for screening and interventions, and optimise the use of evidence to inform policy and program decision-making.

**Methods/Design:**

The study design will use mixed methods to achieve the objectives. Policy, clinical practice and research experts will work collaboratively to develop, test and validate a simulation model of GDM in the Australian Capital Territory (ACT). The model will be applied to support evidence-informed policy dialogues with diverse stakeholders for the management of GDM in the ACT. Qualitative methods will be used to evaluate simulation modelling as an evidence synthesis tool to support evidence-based decision-making. Interviews and analysis of workshop recordings will focus on the participants’ engagement in the modelling process; perceived value of the participatory process, perceived commitment, influence and confidence of stakeholders in implementing policy and program decisions identified in the modelling process; and the impact of the process in terms of policy and program change.

**Discussion:**

The study will generate empirical evidence on the feasibility and potential value of simulation modelling to support knowledge mobilisation and consensus building in health settings.

## Background

Health systems are under continual pressure to provide accessible and effective health services within limited slow growing or reducing budgets. In this context, decisions regarding the best investment of health funds need to be well informed, reviewed regularly and aimed at achieving the greatest health gain for the investment.

The divide between research and health system actions has been frequently recognised [1–3]. Knowledge derived from research and experience will be of little benefit unless it is utilised and its success monitored [1]. There is a need to bridge the gap between the increasingly sophisticated research on using evidence and practitioner knowledge to inform practice and policy and the pragmatic nature of agency decision-making for strategies and actions [2]. Advances in technology have led to increased adoption of tools and methods aimed at integrating diverse evidence sources to inform decision-making [4, 5]. However, rigorous assessment of the value and utility of these methods and tools is required prior to them being more generally adopted for evidence-based decision support. The application of systems science and simulation modelling to the decision-making process is an innovative area with great potential value for those responsible for allocating scarce resources [6].

### What are the challenges of evidence-informed policymaking?

Evidence-informed policy decisions are essential to ensure that health intervention programs and service plans are likely to be effective and offer value for money. However, barriers to the use of evidence to inform decision-making remain [7] and the use of published research to inform policy development is often limited [8]. Descriptive evidence and analytical studies are used to describe issues and inform priorities; however, evidence on the implementation and impact of interventions is less commonly used to inform program planning decisions and strategic actions [7]. In some cases, program decision-making can be driven by “*informed guesswork, expert hunches, political and other imperatives*” [9].

To address this, evidence provided to policymakers needs to be in a form that is useful to them [10–12]. Policymakers require synthesised and localised data that contrasts and prioritises policy options, demonstrates effectiveness of interventions, demonstrates the need for a policy response, demonstrates cost effectiveness of actions, reflects the level of public support for a particular issue and personalises the problem [12, 13]. In addition, policy and program decision-making processes are rarely linear. They are frequently iterative processes and are influenced by a range of inputs such as political environment, budget constraints, resources, values, available expertise and ethics [7, 12, 14, 15].

Even when research evidence is considered, as in public health policy development for the prevention of chronic disease [2], this evidence often points to a large range of risk factors that contribute to the problem, including broader social determinants of health. Our lack of understanding about how these risk factors interact, and which are the most important, have resulted in the development of more comprehensive, cross-sectoral strategies to tackle complex or ‘wicked’ problems [5]. However, this approach may not represent the most efficient or effective approach to reducing disease burden at the population level. Rather, it may act to spread finite resources less intensively over a greater number of programs and initiatives, diluting the potential impact of investment [5].

### Knowledge mobilisation to support evidence-based decision-making

The term knowledge mobilisation (KM) is used to refer to a range of active approaches deployed to encourage the creation and sharing of research-informed knowledge [2]. The number of terms used to describe KM activities is large [16] and have been widely debated. These terms include knowledge translation, knowledge transfer, knowledge to action, knowledge exchange, knowledge interaction, etc. [2]. This multiplicity of terms can be a barrier to clear communication in this field [2]. In this research, the term KM is preferred as it reflects that the process of producing and applying knowledge in the health sector is non-linear and iterative. KM can involve a number of activities, including capacity building, advocacy, implementation, research and evaluation [17]. Not all of these activities are applied in every KM project [17] and they can be applied in different orders; however, they share the common function of generating and sharing research-informed knowledge [2].

KM strategies have been applied to a range of issues, including the quality and effectiveness of health services, addressing policy questions (for example, mapping health inequity and healthcare disparities), and addressing managerial and organisational issues such as the composition of multidisciplinary teams and the costs and consequences of different service models [2, 18]. A key strategy of KM is the production of good quality, synthesised evidence [9] such as scoping reviews, systematic reviews, meta analyses and research summaries highlighting key findings for decision-makers [9, 10].

Traditional methods of KM via evidence synthesis have made a valuable contribution; however, they have a number of characteristics that limit their utility as decision support methods for complex policy questions. Firstly, systematic reviews and meta-analyses focus on clear and specific questions and therefore have a narrow focus of investigation and limited potential to examine complex questions [11, 19]. Secondly, these methods frequently exclude qualitative evidence, and when qualitative evidence is included it is not used to answer the primary research question but only to answer supportive questions such as whether an intervention was acceptable to consumers [19]. Thirdly, these methods produce static overviews of the evidence and policy options that are passively provided to decision-makers, leaving them to interpret that evidence in their localised context and to navigate complexity and uncertainty as they weigh up options for responding to the problem [20].

While there are many KM approaches and techniques, the evaluation of their use is still in its infancy [2, 21]. The limited focus on evaluation of the effectiveness of KM methods, including systems-based ones, has been attributed to the challenges associated with the evaluation task [2], including the methodological challenges of conducting rigorous evaluations. It can be difficult to measure impact, to attribute impact to different strands of the activity in a complex environment, and to minimise the evaluation reporting burden on stakeholders [2].

### Systems approaches to knowledge mobilisation

There are acknowledged synergies between KM and systems science [21]. Systems science methods have emerged as an effective analytical approach with the capacity to examine both complex health problems and the context in which they are embedded [6, 22, 23]. Systems science can be used to map health system components and their interactions; synthesise evidence, examine and compare the potential outcomes of interventions; and guide more efficient investment and conscientious disinvestment of resources [5]. As practical systems-based KM tools and strategies emerge, their efficacy needs to be evaluated and this knowledge to be shared [2, 21].

Systems approaches recognise the highly contextualised nature of health services and communities and, therefore, evidence to inform decision-makers is unlikely to be in the form of prescriptive statements of ‘what works’ [24]. Rather, evidence from a systems-thinking perspective will suggest the range of strategies that will have different types of effects for different groups under certain conditions. Building this type of evidence base will involve undertaking diverse methodologies, including the use of case studies investigating the efficacy of using systems techniques to inform decision-making [24].

Research methods in prevention science have traditionally employed a reductionist approach focusing on the detail of each component of the system. For example, many studies focus on the design, measurement and analysis of specific interventions for specific target groups. These studies have contributed and will continue to contribute significantly to understanding the effectiveness of prevention interventions, gaining knowledge about direct causal relationships and understanding components of complex systems [6, 25]. However, this approach can result in a failure to achieve understanding of the broader system behaviour influencing prevention problems and can hinder insights that may be critical for effective policy and program decision-making [25]. Traditional statistical methods have difficulty accounting for delays between cause and effect, non-linear relationships and unanticipated consequences of interventions [23].

Applying a systems approach through dynamic simulation modelling can provide a method to map, visualise and quantify a complex system, to promote discussion among stakeholders [26], and to identify points of high leverage for intervening. Leverage points are those places in a system where a small shift can create a large impact [27]. Leverage points are difficult to identify in complex systems using traditional reductionist research methods which examine relationships between specific elements of the system in isolation [28–30]. It is also difficult to identify the direction of shift required to obtain the desired outcome without comprehensive analysis and understanding of the system and its behaviour [27, 31]. Unanticipated consequences of interventions can have profound and negative impacts [31, 32], and can lead to policy resistance in which the intended positive impact of the intervention is counteracted by system responses to the intervention itself [32].

Dynamic simulation models allow for rapid integration and use of new evidence for policy analysis, make trade-offs of policy options explicit, and act as a vehicle for advancing controversial, contested and value-laden debates [5, 31, 33]. Their use to explore the implications of policy options can give rise to policy scenarios that have not previously been considered [5].

System dynamics modelling has been used as a tool to represent disease prevalence, risk factors and local context and to simulate the health outcomes of interventions, thus facilitating the alignment of prevention efforts by a range of community stakeholders [34]. For example, Loyo et al. [35] used a stakeholder engagement process to develop a system dynamics model to simulate the impact of various interventions in chronic disease outcomes. The model was used to illustrate which interventions were most effective leverage points in the local context/system and therefore to align and mobilise prevention efforts of community stakeholders [35].

Participatory modelling processes, such as the one described by Loyo et al. [35], provide an opportunity to understand and develop efficient solutions in the health sector [36, 37]. Participatory modelling, firstly, helps community stakeholders understand how multiple variables, factors and interventions interact. Secondly, simulation modelling can test the potential impact of programs and policies in the ‘safety’ of a virtual environment before they are implemented, saving time, effort, costs and resources. Thirdly, modelling demonstrates potential secondary and tertiary effects (and even unintended consequences) of intervention strategies. Fourthly, modelling can guide and prioritise data collection and facilitate dialogue among stakeholders [36].

The process of participatory simulation modelling involves engaging multidisciplinary stakeholders in a group model-building process and can be used in conjunction with a number of modelling methods [31, 37, 38]. The value of this engagement is the development of a shared mental model of the causal pathways and potential intervention points in the system [39]. A participatory modelling approach enhances stakeholder knowledge and understanding of the system and its dynamics in varying conditions. It identifies and clarifies complex and contested real world problems [33] and the impact of solutions, therefore facilitating the development of action statements based on the evidence [39, 40]. The involvement of key decision-makers in the model development and validation increases their sense of ownership and confidence that the model is valid for their local context. They are therefore more likely to draw on the outputs to inform decisions about priority interventions and policies [23, 37, 39, 41].

### Important gaps in knowledge

The application of systems thinking to health improvement is acknowledged as an ongoing challenge [42, 43]. Stakeholder engagement and involvement in the modelling process has been particularly lacking, resulting in unsuccessful projects [42] and a reluctance from ‘non-researchers’ to use models as a decision support tool [33]. A systematic review of the use of simulation modelling to inform surgical patient flow processes found that only half of publications stated that they had produced a model to inform policymakers and health service managers and only 26% actually included policymakers and health service managers in the simulation modelling process [44]. Where policymakers have been included in the simulation modelling process there remains an absence of rigorous analysis of their perspectives on the utility of the model, their learning relating to the development and use of the model, and their commitment to implement the findings of the model [5, 37].

Relationships and collaborations are routinely identified as a key factor in systems approaches [45] and this is particularly true for participatory modelling processes. Important elements for implementing successful systems thinking to address complex issues include the formation of networks and teams, distributed leadership, and strong and effective communication and feedback mechanisms [17]. Understanding the role of participants within the system as well as in the participatory modelling process and bridging professional cultures [45] is key to understanding the factors that will impact on the uptake of simulation modelling as an evidence synthesis tool. Participatory modelling approaches aim to combine multidisciplinary stakeholder perspectives to tackle the social complexity of problems and recognise that different types of knowledge contribute alternative and valuable perspectives to the problem discourse [33].

Evaluation of the participatory simulation modelling process in the health sector has been lacking [5, 41] despite assessment of its efficacy being essential to inform decision-making [5, 37]. Understanding the intricacies of the participatory process [33] and evaluating methods and tools to facilitate participatory modelling is necessary to improve modelling outcomes [4, 31, 37] and further research is required to develop and refine rigorous evaluation methods [39]. The Challenge and Reconstruct Learning (CHaRL) Framework has been proposed by Smajgl and Ward [46] to evaluate participatory modelling processes. This framework can be used for deliberative approaches [47] and involves assessing formalised and facilitated learning among decision-makers and decision influencers at varied policy levels. The key component of the CHaRL framework is the change in perception or belief about assumed causality within the system. In other words, participants’ mental models are challenged by the presentation of different perspectives, scientific evidence and system interactions through the modelling process. The change in mental model can be measured using individual value and attitude/belief orientations recorded by participants pre- and post- the modelling process [46].

### Study objectives

The objectives of the research are to apply and evaluate a simulation modelling approach, using gestational diabetes as a case study to:

1. Pilot simulation modelling to optimise the use of evidence to inform policy and program decision-making by synthesising and integrating diverse evidence sources into a dynamic simulation model of gestational diabetes using a participatory modelling approach. The model will be used to understand the complex interrelation of factors that drive gestational diabetes mellitus (GDM) rates and test options for interventions.
2. Investigate the perceived value and efficacy of participatory simulation modelling methods as an evidence synthesis and decision support method in an applied health sector context.

### Using GDM as a case study

GDM is a complication of pregnancy that is defined as carbohydrate intolerance resulting in hyperglycaemia (abnormally high blood sugar) of variable severity with onset or first recognition during pregnancy [48]. GDM defined in this way includes women with undiagnosed pre-existing diabetes, as well as those for whom the first onset is during pregnancy (especially during the third trimester of pregnancy). The prevalence of GDM is increasing both in Australia and internationally [49].

Identified risk factors for GDM include maternal body mass index of at least 30 kg/m^2^ [50–52], increasing maternal age [52], physical inactivity [50, 52], increasing parity, and ethnicity [53]. Women are also at increased risk if they have a history of GDM [52], previously had a macrosomic baby (birthweight greater than 4000 g), a family history of diabetes [52], polycystic ovary syndrome [52], or a diet low in fibre [54, 55].

Perinatal risks associated with GDM include macrosomia, shoulder dystocia, other birth injuries, hypoglycaemia and perinatal mortality [53, 56]. Long-term risks for the infant from GDM include sustained impairment of glucose tolerance [57], subsequent obesity [58] (although not when adjusted for size) [59], and impaired intellectual achievement [60]. For women, gestational diabetes is a strong risk factor for the development of diabetes later in life [61, 62].

Although the risks associated with gestational diabetes are well recognised, debate remains as to whether screening and treatment to reduce maternal glucose levels reduce these risks [53, 63]. Given this uncertainty, professional groups disagree on whether to recommend routine screening, selective screening based on risk factors for gestational diabetes, or no screening [53]. There is also debate over the efficacy of using a single raised blood glucose result to diagnose GDM [63].

The Australian diagnostic threshold for GDM was changed to be consistent with WHO criteria from January 1, 2015. The WHO report from which the criteria were obtained acknowledges that the evidence for the threshold chosen is weak. However, they argue that the benefits of treatment, i.e. reduction of risk for macrosomia, shoulder dystocia and pre-eclampsia is sufficient justification. Treatment of gestational diabetes once diagnosed is generally medicalised (insulin treatment) and involves intense use of health services, mostly in the third trimester. Investigations of the cost implications of using the lowered diagnostic threshold concluded that cost effectiveness will only be achieved if treatment reduces the risk of caesarean section birth and developing Type 2 diabetes mellitus [64, 65].

Pregnancy has been identified as a point in the life cycle where individuals have increased motivation to commit to health improving behaviours, for example, in smoking cessation [66]. A diagnosis of GDM (or even a glucose tolerance test result that approaches the diagnostic cut-off) may provide a powerful leverage point for multidisciplinary health interventions promoting lifestyle change to reduce the risk of developing diabetes later in life. Almost all women (95%) with a diagnosis of borderline GDM in an Australian study identified that managing their borderline GDM was important or very important for the health of their baby and themselves [67]. Enablers identified by women to implement lifestyle change during pregnancy include family support [66, 67], physical access to programs, knowledge (about diet, exercise and GDM), and motivation levels [67].

Previous models of GDM developed to investigate the cost effectiveness of screening and treatment regimens [64, 65, 68, 69] have provided valuable evidence to inform decision-making. However, these models focussed on an economic evaluation of specific treatments and did not analyse the wider outcomes of policy and program decisions, including the intended and unintended consequences and resource implications of interventions delivered in the health system [70]. Dynamic simulation modelling has been used to investigate the intergenerational impact of GDM on the development of Type 2 diabetes mellitus among First Nations and other population groups in Canada [71]. This model included representations of factors contributing to the development of diabetes mellitus, including changes in behaviour regarding diet and physical activity over time and found that GDM disproportionately contributed to the development of Type 2 diabetes mellitus in First Nations populations compared with other population groups [71].

Dynamic simulation modelling provides an opportunity to explore and compare the implications of health intervention options for GDM services in the Australian Capital Territory (ACT) and to inform policy and program decision-making. The simulations derived from the model can be used to explore the dynamic interaction of risk factors such as maternal weight and weight gain (pre and during pregnancy); the impact of screening earlier or later in pregnancy; the impact of universal or selective screening; the impact of lowering the diagnosis threshold on the number of women diagnosed, health outcomes and health system impacts; the implications of intervention options for prevention and treatment of GDM with different target groups and with different timings (e.g. at the start of pregnancy, during pregnancies, between pregnancies); GDM diagnosis and risk of later development of Type 2 diabetes in the ACT; and the short- and long-term outcomes for mother and baby following treatment for GDM.

The current research project will contribute to knowledge on the application of systems thinking to a localised health system case study by undertaking, validating and evaluating a participatory simulation modelling process focusing on GDM.

## Methods/Design

### Design overview

The study design will use mixed methods to achieve the research objectives. A participatory simulation modelling approach will be used to synthesise evidence and explore strategies for GDM diagnosis, early intervention and management (Objective 1). Evaluation of the modelling process as a systems-based knowledge synthesis tool will incorporate both qualitative and quantitative methods (Objective 2).

### Research questions

Simulation modelling will be used to answer the following research questions about GDM interventions in the ACT. Model simulations will explore:

- The dynamic interaction between risk factors such as pre-pregnancy maternal weight, maternal weight gain during pregnancy, GDM diagnosis and life-time risk of developing of Type 2 diabetes for mothers and babies in the ACT
- The short- and long-term outcomes for mother and baby following treatment for GDM in the ACT
- The impact of changing the diagnosis threshold on the number of women diagnosed, health outcomes and the health system impacts (including health economic analysis)
- Health outcomes achieved from priority interventions identified by participants
- Cost effectiveness of priority interventions identified by participants

This research will also explore the effectiveness of participatory simulation modelling methods to optimise the use of evidence to inform policy and program decision-making through qualitative and quantitative methods investigating the participatory modelling process and evidence of impact on decision-making (detailed further below). The specific questions to be answered by this research include:

- Whether simulation modelling is an effective tool to facilitate evidence-informed decision-making in an applied health setting
- The efficacy of applying a participatory approach to model development
- The benefits and limitations of using simulation modelling to explore potential outcomes from a range of policy and intervention options to inform decision-making

### Study setting

The study is being conducted as part of an ongoing initiative of The Australian Prevention Partnership Centre to apply systems approaches to the prevention of chronic disease. The research will be carried out at the ACT Government Health Directorate, which provides publicly funded health services for a population of approximately 390,000 in the ACT and is the major health referral centre for the Greater Southern Region of NSW. The total catchment area population is over 600,000 people. Tertiary level maternity services are provided by Canberra Hospital at the Centenary Hospital for Women. There are two publicly funded hospitals and one private hospital in the ACT, providing maternity services.

The number of women giving birth in the ACT is over 6000 per year. Approximately 15% of these women are not ACT residents but access services in the ACT for high risk pregnancy complications (i.e. requiring tertiary level care). There a number of models of antenatal maternity care provided in the ACT including hospital-based out-patient care, tertiary level care, private midwifery care, and shared care (which is integrated with primary healthcare providers).

A specialist gestational diabetes service with satellite clinics in community health centres works with generalist maternity services to provide education and health services for women with gestational diabetes.

### Participants

Purposive sampling will be used to recruit participants with a range of expertise such as endocrinology, obstetrics, neonatology, diabetes education, nursing, midwifery, policy, health economics, exercise physiology, pathology, public health, research, allied health, health service management, consumers (healthcare recipients) and the simulation modelling expert team. The anticipated number of participants is 10 to 15 to allow for wide engagement with influential leaders while maintaining a manageable dialogue with meaningful contributions from all members.

The inclusion criteria for participants is that they are recognised experts in providing care, planning services, undertaking research or developing policy for the diagnosis and management of GDM. Participants must also be willing to attend model development and application sessions and participate in the evaluation.

Participants in the group model building and model validation processes will be asked to provide written consent prior to participating.

### Procedure

#### Objective 1 – Participatory model development

##### Model development

This research will employ a participatory simulation modelling process, which will involve the following steps [4, 26, 31, 36]:

- Forming an expert sub-group of the participants listed above who will define the boundaries of the model. A model is not able to include in detail every possible factor, relationship and intervention, and therefore only those that are relevant to the policy and practice questions to be answered by the model should be included in the first instance. Engaging with the literature and collaborating with stakeholders and researchers to understand the risk factors for GDM, options for GDM diagnosis and intervention, and reach agreement on the priority health and economic outcome indicators to be included in the model structure
- Identifying data sources and populating the model with data (parameterising the model)
- Deciding which local and/or national data on current practices and behaviours should be incorporated into the model
- Identifying potential intervention leverage points and mapping the mechanism by which interventions have their effect in the model
- Validating the model using accepted validation methods such as assessment of face validity, system behaviour reproduction, parameter estimation, sensitivity analysis and statistical testing [41]
- As the model develops into a functioning simulation tool, exploring possible scenarios and prediction of outcomes
- Ensuring the purpose, assumptions and limitations of the model are clearly stated
- Using the final model to explore the timing, frequency and combination of interventions that deliver optimal impact

The participatory model development process will identify the factors to be represented in the model. It is anticipated that a combination of high level aggregated, individual characteristics and interactions and event-based factors (e.g. service utilisation), will be identified. Therefore, a more flexible hybrid modelling approach will be adopted incorporating system dynamics, agent-based and discrete event modelling methods.

System dynamics modelling methods were created in the 1950s by Jay Forrester in the field of engineering. System dynamics modelling utilises feedback loops (causal loop diagrams) and stock (accumulations) and flow diagrams to represent complex systems [6, 23, 72]. This modelling method represents the dynamics of the system at a high level of abstraction [6], making them an efficient form of modelling in terms of computing resources. System dynamics simulates patterns and trends in system behaviour. Simulation experiments can be used to compare and contrast intervention alternatives to inform decision-making [70].

Agent-based modelling (ABM) methods have been developed more recently and allow for representation of individuals or agents within the system. The model can be built from the ground up by defining agents, their behaviours and their interactions [6, 72]. ABM is a computational method used to examine the actions of agents (e.g. individuals) situated in an environment (e.g. neighbourhood). ABMs specify decision rules controlling dynamics such as ‘If–Then’ statements and mechanistic interactions among agents. When the program is run, agents interact with one another and their environment, often resulting in counterintuitive insights about behaviour of agents and the system [23]. Incorporating ABM components allows flexibility to incorporate the dynamics of people making decisions affecting population health outcomes, and thus efficient planning of healthcare interventions [70].

Discrete event modelling methods represent the system as a process, namely, as a sequence of operations or events performed across entities [72]. For example, discrete event methods are frequently used to represent and improve efficiency of health services such as emergency departments. This modelling method represents complex systems at a low level of abstraction. The core concepts in discrete event simulation (DES) are events, entities, attributes and resources. An event happens at a certain time point in the environment and can affect resources and/or entities. Entities have attributes and consume resources while experiencing events, but consumption is not affected by individual-level behaviour. Attributes are features or characteristics unique to an entity. They can change over time or not. Resources are objects that provide a service to an entity. Queues are another important concept in DES and occur when several entities compete for a specific resource for which there is a constraint [70]. DES modelling is useful to analyse resource utilisation, throughput of services and the impact of varying policy decisions [70].

Advances in modelling software technology now enable multiple modelling methods to be integrated [72]. This allows for modellers to represent the many interacting components of a system and the complex interplay between individual behaviour and social connections across populations [6].

##### Model application

Once the model develops into a functioning simulation tool it will be used to explore possible scenarios and prediction of outcomes. During this phase, a broader stakeholder group will be formed and engaged in policy/strategy dialogues facilitated by interaction with the model and explore the costs and benefits for a range of intervention options. The composition of the stakeholder group will include the full scope of disciplines and consumers outlined in the Participants section. The model application process aims to refine the model as well as to demonstrate the utility of the model to key decision-makers so as to inform policy action and program decisions.

The transdisciplinary simulation modelling process provides an opportunity to establish network relationships and analyse policy and program options based on outcomes simulated. An action statement regarding GDM diagnosis and treatment in the ACT based on the simulation modelling work and synthesised evidence will be developed with the expert group.

##### Data analysis

The model will be built using AnyLogic^®^ 7.2, St Petersburg, Russian Federation. AnyLogic^®^ software allows for multiple modelling methods to be integrated into a single hybrid model providing participants both flexibility and transparency in model design.

Model parameterisation involves populating the model with data and will evolve in accordance with the participatory modelling process. This will make use of the following:

- Secondary analysis of de-identified administrative data to inform transitions (hazard rates/probabilities/relationships between risk factors) within the model structure. For example, regression analyses may be conducted to determine the contribution of gestational diabetes in relation to other risk factors to perinatal outcomes such as birthweight
- Published demographic information such as age and gender characteristics, age-specific fertility rates, population estimates of weight status categories
- Published results from research on intervention effects such as the impact of targeted pregnancy weight management programs focused on nutrition or physical activity on the development of GDM
- Local expert knowledge to supplement available data
- Partitioned administrative and/or available survey data to calibrate the model

Statistical analysis of administrative data will be conducted using IBM SPSS Statistics version 22, United States.

Data availability is a potential limitation to this study. It is proposed that, where data is not of high quality or is not available, placeholder values will be used and tested using the following methods. Firstly, the model simulations will be analysed against trends and patterns observed in historical data and, secondly, sensitivity testing will be conducted around the missing values to determine if the model outputs depend significantly on them. When parameters are identified that the model is sensitive too, this can be used to guide and prioritise future research activities to obtain these important pieces of data. Assumptions surrounding the use of placeholder values will be made explicit in descriptions of the methods used to develop the model.

Validation of the model is necessary to assess the logic, soundness and utility of the model outputs [41]. Validation of the model can be conducted as part of the model development process by conducting tests and involving the model users in assessing the validity of the model [73].

The model will be validated using accepted validation methods such as assessment of face validity, system behaviour reproduction, parameter estimation, sensitivity analysis and statistical testing [41]. Expert participants in the model development process will be asked to assess whether the model and its behaviour and outputs are reasonable given their knowledge of the system [73]. The model behaviour will also be tested against historical data and model simulations over time will be assessed. Available data will be partitioned with a subset used to build the model and the remaining data used to determine (or test) whether the model replicates the historical system behaviour [73]. Parameter variability and sensitivity analyses will also be conducted to test model behaviour and to determine which parameters the model is most sensitive too. Those parameters that are sensitive, that is they cause significant changes in the model’s behaviour or output, should be made sufficiently accurate prior to using the model [73].

#### Objective 2 – Evaluation of a participatory approach to dynamic simulation model building

##### Procedure

The case study methodology allows for investigation of the strengths, weaknesses and evaluation of participatory simulation modelling as a mechanism to influence policy and program decision-making and develop action statements [2]. Little is known about the value, strengths and limitations of simulation modelling as applied to ‘real world’ health policy decision-making. The key research questions addressed in this study include those relating to engagement of experts in the process; perceived commitment, influence and confidence of stakeholders in implementing policy and program decisions identified in the modelling process; and measuring the impact of the process in terms of policy and program change.

The evaluation of the participatory modelling process is informed by the CHaRL Framework proposed by Smajgl and Ward [46]. The CHaRL framework can be used for deliberative approaches and involves assessing formalised and facilitated learning among decision-makers and decision influencers at varied policy levels. The key component of the CHaRL framework is the change in perception or belief about assumed causality within the system. In other words, participants’ mental models are challenged by the presentation of different perspectives, scientific evidence and system interactions through the modelling process. The change in mental model can be measured using individual value and attitude/belief orientations recorded by participants before and after the modelling process [46].

Therefore, the evaluation methods to determine the effectiveness and impact of systems dynamic modelling will include investigating the:

- Participation in the process, e.g. response rate to invitations, attendance and retention at modelling sessions and subsequent deliberative forums
- Participants’ perceptions of the key factors that contribute to GDM and the best use of resources to diagnose and manage GDM through survey responses
- Group interactions, contributions and engagement with the process by qualitative analysis of audio recordings of the model building and engagement sessions
- Informant views via semi-structured interview on the:
  - value of simulation modelling as an evidence synthesis tool
  - strengths and limitations and intention to use simulation modelling in the future
  - perceived enablers and barriers to the use of simulation modelling
  - personal response to the participatory modelling process
- Follow-up environment scan to determine policy and program decisions that were informed by the modelling process and the model outputs

##### Data analysis

Quantitative analyses will include measuring and reporting the number of sessions attended, and analysing the responses recorded on the before and after forum surveys.

Participants will be asked to record their views on the main contributing factors to GDM, the optimal time for screening for GDM and how they would allocate resources to a hypothetical new service for women with GDM. They will also be asked to provide self-reported evaluation feedback reflecting on their learning and ways to improve the modelling sessions.

Qualitative analyses will include analysing the data collected during:

1. Model development sessions
2. Model application sessions
3. Semi-structured interviews (pre- and post-modelling workshops)
4. Notes and memos based on meetings and de-identified conversations with participants and the modelling team

The model development and application sessions will be audio recorded, primarily to allow the investigators to review content information and expert advice provided by participants relating to model development. The recordings, participant observations and field notes will be kept to highlight particularly valuable comments and analyse behaviours or interactions between participants. The analysis of field notes will be triangulated against the audio recordings and interview transcripts.

Semi-structured interviews will be conducted with participants of the model development and model application sessions. Participants will be purposively selected for interviews to provide a range of perspectives and interviews will be conducted face-to-face where possible. The main domains to be covered will include participant’s perceptions or ‘mental model’ of GDM through the modelling process, value of simulation modelling as an evidence synthesis methodology to inform decision-making, and intention to use this method in the future. The proposed interview questions are contained in Box 1.

Field notes relating to meetings and informal discussions will be maintained by the researcher in a journal format and will be included in the qualitative data analysis.

>Audio recordings will be transcribed and integrated with field notes and reflections. Transcriptions will be de-identified, collated and coded so that only general themes emerge.

Interview data will be independently coded by two investigators. Initial codes will be derived from the research aims and subsequently refined over two coding cycles. The two coders will compare and agree upon codes and emerging themes at the end of each cycle, resolving disagreement by consensus opinion or by the creation of new, mutually agreeable, codes/themes.

Data analysis will be iterative and begin with identifying central organising concepts, patterns and themes from the coded data. Thematic analysis will be reflective and revised by revisiting the coded and collated data to ensure that identified themes and subthemes are coherent, distinctive and relevant to the research question [74].

Common and repeated themes identified from the modelling sessions will be investigated through interviews to better understand informant views in relation to specific topics, and to assess the strength and importance of various themes. A comparative analysis will be conducted to understand the range of participant views in relation to their role perspective and level of power within their organisation, e.g. clinician, researcher, manager and policymaker views.

This research involves investigators who currently work within the local health sector. This provides some advantage as these investigators have good knowledge of the system and context; however, it also presents challenges and limitations. For example, the investigators’ willingness to identify and report on system limitations may be impacted by their professional affiliation with the organisation. The involvement of external co-investigators and the use of independent reporting mechanisms through The Australian Prevention Partnership Centre are mitigation strategies to be employed for this challenge. The use of voluntary recruitment processes and confidentialised analyses of individual input and participation will be employed to address perceptions of coercion or concerns of repercussion from either participating or declining to participate in this research.

A follow-up environment scan to determine policy and program decisions that were informed by the modelling process and the model outputs will be conducted three to 3–6 months after the model engagement workshops. This will involve interviews with end users and document analyses to determine the use of model outputs to inform decision-making.

#### Data storage and management

All audio-recorded data from the model development and model application sessions will be de-identified by using codes instead of names and removing any potentially identifying text from transcripts. Data will be stored securely on password protected computers or ACT Health secure servers and will only be accessible to the researchers.

Paper surveys will be anonymised and scanned to create an electronic file to be stored in secure folders on a secure server only accessible to the researchers. The paper surveys will then be securely destroyed. Clinical and administrative data to be used for the project will be de-identified prior to analysis.

## Discussion

This project will apply systems science and simulation modelling to GDM in the ACT as a case study.

The outcomes will include, firstly, producing a model that will be a functioning simulation tool to explore possible scenarios and the impact of those scenarios on health outcomes for the mother and baby as well as service impacts for the health system; secondly, developing a joint commitment for policy action and program decisions through engagement with the stakeholder group and, thirdly, evaluating the use of simulation modelling to inform decision-making.

The participatory model-building process will be informed by a multidisciplinary expert stakeholder group. This provides an opportunity to ensure the model reflects the shared understanding of the causal pathways and potential intervention points in the system.

Simulation modelling methods will be used to explore and compare strategies for GDM diagnosis, early intervention and management. The modelling will include interaction between risk factors, the short- and long-term outcomes for mother and baby, and potential modes and timing of intervention.

Importantly, involving key decision-makers and experts in the model development and validation process increases the acceptability of the model for the local context. The model is therefore more likely to be useful to inform decisions about priority interventions and policies.

Systems science is emerging as an effective way to examine both complex health problems and their context. It can be used to synthesise evidence, examine and compare potential outcomes of policy options, and guide the best use of limited resources through methods such as simulation modelling. This research will contribute to existing knowledge, firstly, by applying a participatory process to simulation modelling in a local health setting; the participatory process will engage expert stakeholders in the development of a functioning model to inform decision-making. Secondly, by developing and incorporating evaluation methods to investigate the efficacy of simulation modelling as an evidence synthesis tool. Thirdly, by using quantitative data to develop a simulation model to inform health policy and program decisions.

## Box 1 Semi-structured interview questions to obtain key informant views

Prior to workshops

 Based on your experience, what are the current challenges that GDM services are facing? What do you think is driving these challenges? What changes do you think GDM services need to make to cope with these challenges? Which interventions would you prioritise to prevent and manage GDM?

 Could you talk a little about your thoughts on evidence-based decision-making in the health policy context? To what extent do you think evidence is used to inform health policy and program decisions? What factors have you found to be useful to support its use? What are the main challenges?

 Have you had experience using results of evidence synthesis methods such as systematic reviews, meta-analyses? Did they meet your needs for evidence to inform your decision-making? From your experience, what are the strengths and limitations of these methods? What other forms of evidence do you use in decision-making?

 Have you participated in any form of simulation modelling process before? (If reply yes) Could you tell me about the modelling process and your experience of it? In your opinion, what are the benefits and limitations of simulation modelling as an evidence synthesis tool?

Post workshops

 Could you tell me about your experience of participating in the simulation modelling process? What are the strengths and weaknesses of simulation modelling as an evidence synthesis tool?

 Has/How has the modelling process influenced your opinion of the key factors that contribute to GDM? Has/How has the modelling process influenced your opinion of the best use of resources to screen for and treat GDM?

 Will you use the outcomes of the gestational diabetes modelling process to guide your future decision-making? Why or why not?

 Based on your experience would you say simulation modelling is worthwhile for health sector policy/practice settings? Why/why not? Do you intend to use the outputs of this model or participate in other simulation modelling projects in the future? Why or why not?

 In your opinion, what would you say are facilitators and barriers to the use of simulation modelling to synthesise evidence for decision-making?

 Do you have any recommendations to improve the process for using simulation modelling as an evidence synthesis tool?

## Abbreviations

ABM: agent-based modelling
ACT: Australian Capital Territory
CHaRL: Challenge and Reconstruct Learning
DES: discrete event simulation
GDM: gestational diabetes mellitus
KM: knowledge mobilisation
